# An Unusual Presentation of Acute Severe Mitral Regurgitation

**DOI:** 10.7759/cureus.63271

**Published:** 2024-06-27

**Authors:** FNU Arty, Sai Rakshith Gaddameedi, Malay Rathod, Hamzeh Nasr, Pratik Panchal, Anthony Ricca

**Affiliations:** 1 Internal Medicine, Monmouth Medical Center, Long Branch, USA; 2 Medicine, Monmouth Medical Center, Long Branch, USA; 3 Cardiology, Monmouth Medical Center, Long Branch, USA

**Keywords:** mitral valve replacement, pulmonary edema, transthoracic echo, cordae tendinae rupture, acute mitral regurgitation

## Abstract

Mitral regurgitation (MR) results from retrograde blood flow from the left ventricle to the left atrium. Common etiologies of acute severe MR include papillary muscle rupture from myocardial infarction, leaflet perforation in infective endocarditis, chordal rupture (pop) in myxomatous valve disease, acute rheumatic fever with carditis, or functional MR due to cardiomyopathies, myocarditis or Takotsubo cardiomyopathy. Here, we present an unusual case of acute severe MR due to ruptured chordae tendineae likely secondary to degenerative valve disease.

A 59-year-old male with a past medical history of hypertension and renal calculi was evaluated in the outpatient office for a urologic procedure. He was sent to the emergency room with left-sided chest pain, 6/10 in intensity, burning in nature, and non-radiating with no aggravating and relieving factors. He had nausea and vomiting for the past three days. He reported similar chest pain at rest and on exertion multiple times over the past year. He also had a chronic cough with no recent changes. His examination was unremarkable. Chest X-ray showed interstitial lung markings. Electrocardiography revealed an old right bundle branch block, but no ST/T-wave changes. He was admitted and treated for atypical pneumonia with ceftriaxone and azithromycin. The following morning, he complained of persistent chest pain 9/10 in intensity which improved with nitroglycerin. His examination revealed a new onset holosystolic murmur heard over the precordium. A two-dimensional echocardiogram showed a preserved ejection fraction of 55-60%, severe MR with eccentric jet, concerning for partially flail leaflet of the mitral valve. He was transferred to the university hospital for mitral valve replacement.

Patients with acute rupture of chordae tendineae usually progress to severe mitral valve regurgitation. These patients usually present with pulmonary edema, signs of heart failure, and cardiogenic shock. Papillary muscle dysfunction, as well as partial or complete rupture of the mitral chordae can be detected as a new-onset holosystolic murmur and can be a crucial sign for early recognition. In our case, the patient developed a new holosystolic murmur on day two of admission which was recognized early, and prompt surgical intervention was performed.

## Introduction

Mitral regurgitation (MR) results from retrograde blood flow from the left ventricle to the left atrium. It is present in 2% of the population [1]. There are multiple etiologies of MR, including acute rupture of cordae tendineae. Since Corvisart’s 1806 description of chordae tendineae rupture, it has been increasingly noted as a significant cause of MR. Patients with acute rupture of chordae tendineae usually develop severe mitral valve regurgitation. These patients deteriorate quickly with sudden-onset cardiogenic shock and pulmonary edema causing hemodynamic instability that necessitates prompt medical and often surgical intervention. We present a rare case of asymptomatic acute severe MR due to ruptured chordae tendineae.

## Case presentation

A 59-year-old male with a history of hypertension and renal calculi presented to the emergency department with symptoms of nausea, vomiting, headache, and non-radiating left-sided chest pain. The chest pain was described as a burning sensation, rated 6/10 in intensity, with no specific aggravating or relieving factors. The patient reported an unremarkable coronary catheterization seven months before this admission. He also reported a chronic dry cough with no recent changes. Physical examination in the emergency department did not reveal any significant abnormalities and his vital signs were stable. Chest X-ray showed diffuse interstitial markings and the electrocardiogram indicated normal sinus rhythm with an old right bundle branch block without ST-segment changes. Pertinent laboratory findings revealed mildly elevated high-sensitivity troponin levels and B-type natriuretic peptide levels, as shown in Table 1.

**Table 1:** Troponin and B-type natriuretic peptide levels.

Parameter	Patient’s results	Normal range
Troponin	0.111 ng/mL	0.02–0.045 ng/mL
B-type natriuretic peptide	240 pg/mL	<100 pg/mL

**Figure 1:**
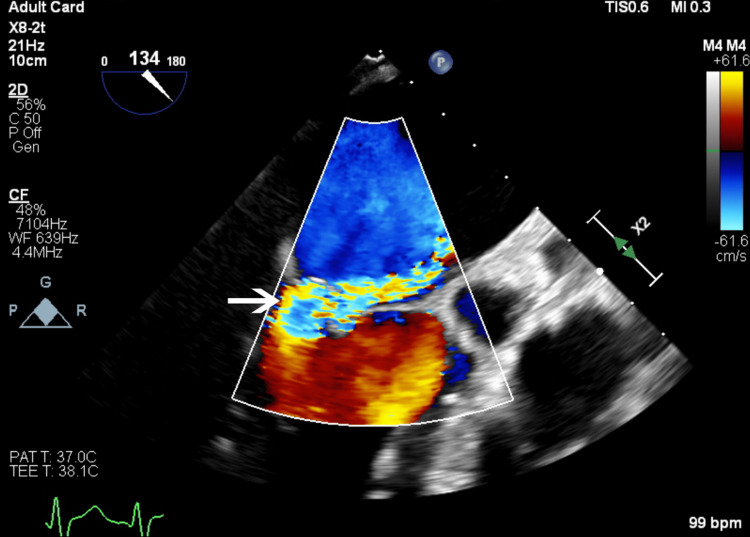
Transesophageal echocardiography image demonstrating a two-dimensional view of mitral regurgitation jet in color Doppler.

**Figure 2:**
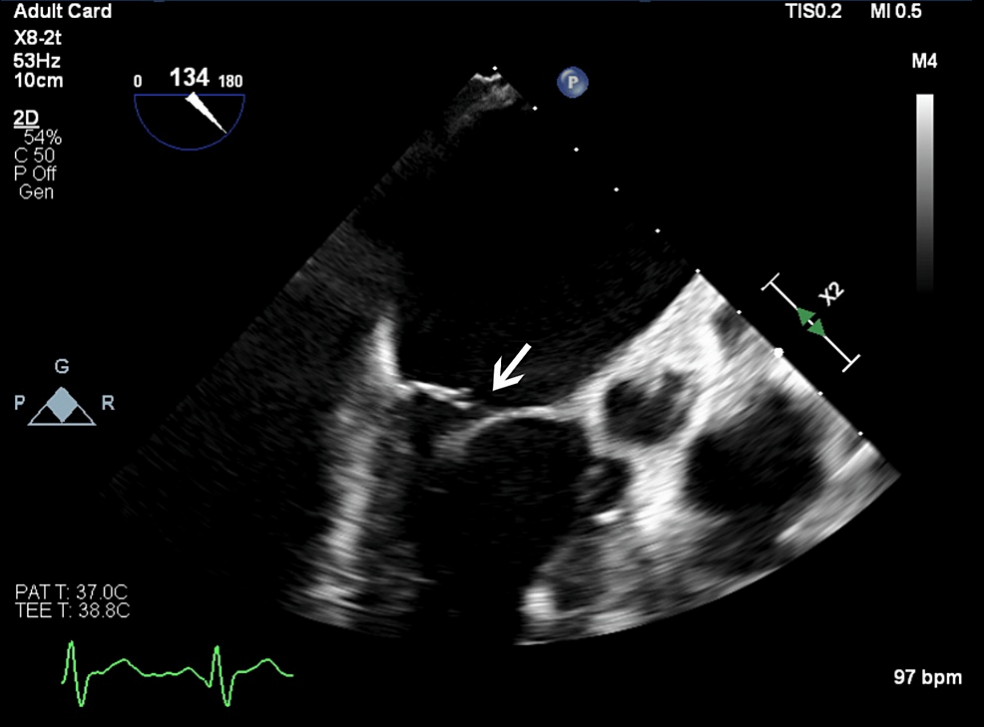
Transesophageal echocardiography image highlighting rupture of the posterior leaflet of the mitral valve causing mitral regurgitation.

**Figure 3:**
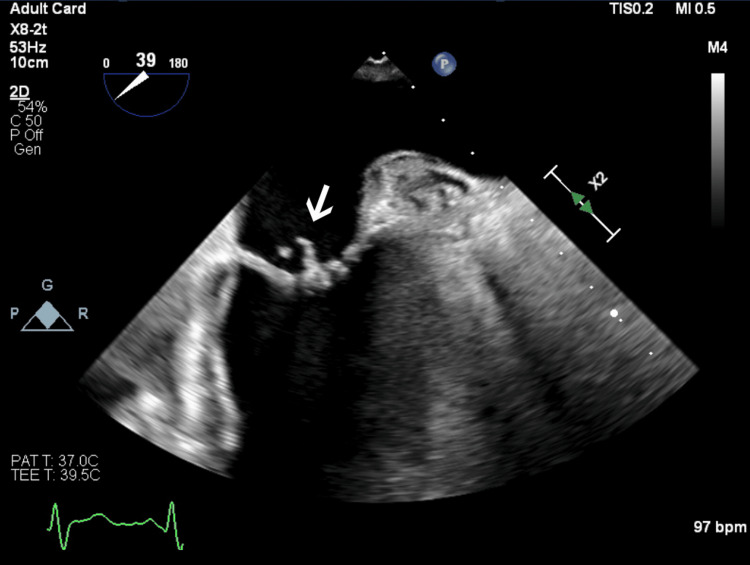
Transesophageal echocardiography image of the patient’s mitral valve clearly demonstrating posterior leaflet prolapse with a ruptured chordae tendineae.

In the emergency department, ceftriaxone 1 gm daily and azithromycin 500 mg daily were given for the treatment of possible atypical pneumonia. His known hypertension was managed by carvedilol 3.125 mg twice daily, valsartan 320 mg daily, and hydrochlorothiazide 25 mg daily and continued as an inpatient. The following morning, he reported persistent chest pain 9/10 in intensity which was unresponsive to nitroglycerine 2% ointment and nitroglycerin tablet 0.4 mg. His physical examination revealed a new-onset 3/6 holosystolic murmur heard over the precordium. Urgent transthoracic echocardiography (TTE) revealed severe MR due to a partially flail posterior leaflet with normal ejection fraction and a mildly dilated left atrium. The next day, transesophageal echocardiography (TEE) was performed to rule out infective endocarditis and confirmed severe MR (Figure 1) and ruptured chordae tendineae with a flail posterior leaflet with no vegetation (Figures 2, 3).

**Figure 4:**
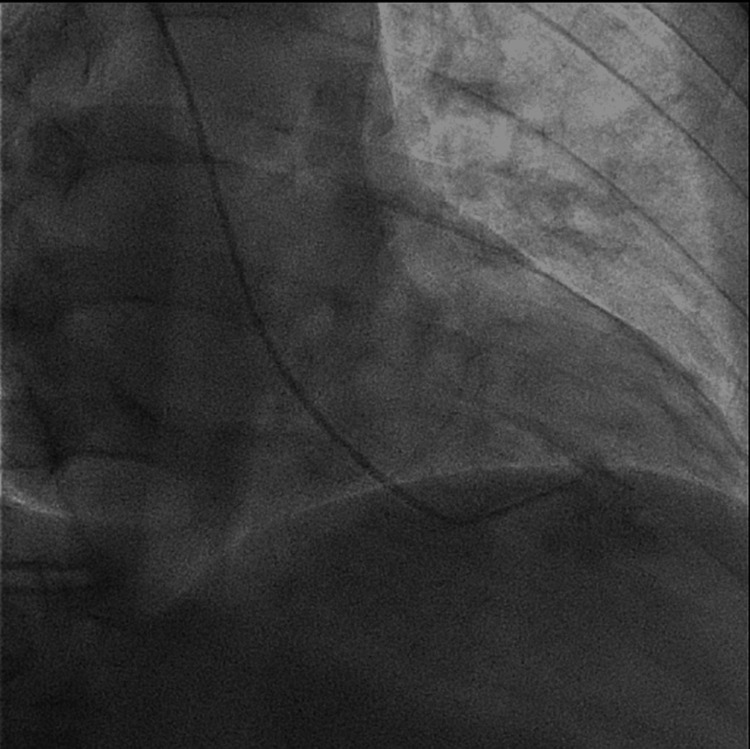
Left ventricular systolic mitral regurgitation on angiography.

**Figure 5:**
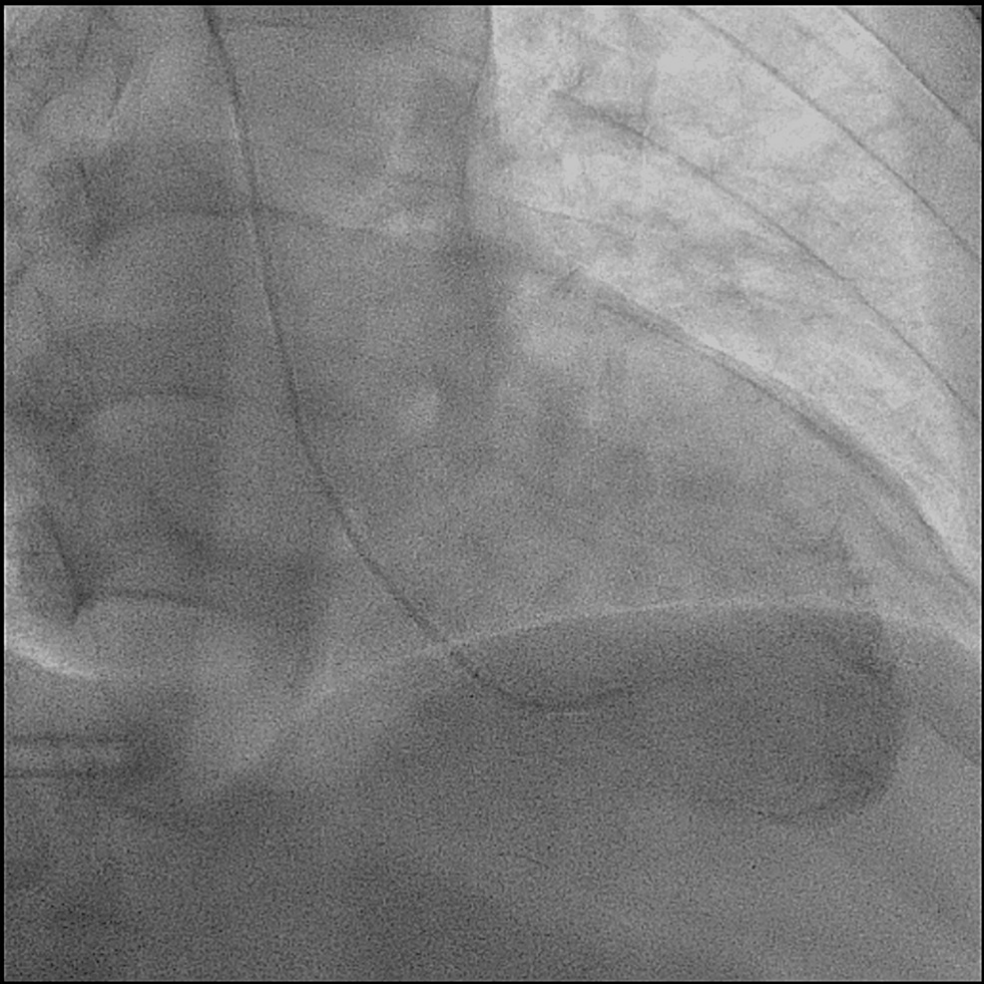
Left ventricular diastolic mitral regurgitation on angiography.

Cardiac catheterization was performed to rule out ischemic etiology which showed mild coronary artery disease with severe MR (Figures 4, 5).

The patient was immediately transferred to the university hospital for urgent mitral valve replacement, which involved a mini-thoracotomy and bio-prosthetic mitral valve replacement. The patient’s pathology report revealed degenerative valve disease with mild myxomatous mitral valve, which was likely the cause of severe MR. The patient developed atrial fibrillation postoperatively which was controlled with metoprolol. He was discharged on metoprolol 25 mg two times a day, Eliquis 5 mg two times a day(for atrial fibrillation), and colchicine 0.6 mg daily for post-pericardiotomy syndrome prevention. On a follow-up visit one week later, he was stable with rate-controlled atrial fibrillation and no new complaints.

## Discussion

MR is the most common valvular heart disease followed by aortic stenosis. Prevalence of MR increases with age, involving <1% of people below the age of 55 while 9% of people aged 75 or older. MR usually results from papillary muscle rupture, chordal rupture, or leaflet perforation which can occur either due to primary mitral valve diseases, such as infective endocarditis, mitral valve prolapse (2-3% of the population), rheumatic valve disease, amyloidosis, or secondary to left ventricular abnormalities such as ischemia or dilated cardiomyopathy [2]. Infective endocarditis causes leaflet perforation or chordal rupture, mitral valve prolapse causes spontaneous chordal rupture due to myxomatous degeneration, and myocardial infarction, especially inferior wall myocardial infarction, causes papillary muscle rupture due to loss of blood supply. It was reported more recently that chronic atrial fibrillation can cause annular dilatation, which causes MR, and atrial cardiomyopathy. Medically treated patients with this illness have a high annual mortality rate of 6.3% [3].

There are differences in the pathophysiology of acute and chronic MR. In chronic MR, the left ventricle dilates to accommodate regurgitant volume. This adaptation preserves cardiac output and stroke volume. While left ventricular diastolic pressure remains the same, left atrial size and compliance increase to accommodate regurgitant blood volume. Acute MR is a cardiac emergency that presents as sudden-onset pulmonary edema and heart failure and progresses to cardiogenic shock [4,5]. In acute MR, left ventricle size and compliance remain the same due to lack of time to adapt to acute changes, resulting in increased backflow of blood to the left atrium and decreased stroke volume. Left atrial size remains the same which results in increased left atrial pressure and subsequently increased pulmonary capillary wedge pressure and pulmonary edema.

In adult males, mucoid degeneration of the mitral valve or chordae tendineae is the main reason for its rupture. It mainly involves posterior leaflet (54%), anterior leaflet (36%), or both (10%). According to studies, the cross-sectional area of the collagenous core decreases, and the cushions of connective tissue that form beneath the endocardium increase with age. These two factors may eventually cause the chordae to stretch and rupture. According to earlier research, the posterior mitral leaflet is more commonly involved (54-79%). According to Sedransk et al., the posterior leaflet chordae and marginal chordae are thinner than the basal and anterior leaflet chordae. This thinness increases the chordae’s susceptibility to stress and strain as well as the likelihood of rupture [6]. Patients usually present with pulmonary edema (16%). In one study, dyspnea was found to be a frequent clinical presentation among patients (74%), especially in primary chordae tendineae rupture. Asymptomatic presentation was rare, seen in only 4% of patients [7].

The initial diagnostic test of choice for acute MR is TTE to evaluate the severity of MR, left and right ventricular function, and mechanism of MR. Medical management includes vasodilator therapy such as nicardipine or sodium nitroprusside to decrease afterload which increases stroke volume and decreases the backflow of blood to the left atrium from the left ventricle. An intra-aortic balloon pump can also be used. Mitral valve surgery is the treatment of choice for acute MR. Mitral valve repair is better than replacement owing to fewer complications, lower operative mortality, and longer survival [8,9]. Early surgery in asymptomatic patients improves clinical outcomes and successful repair rates of 80-90%[10].

In our case, the patient presented with non-specific symptoms which were thought to be pneumonia due to normal cardiologic examination findings on admission. However, he was found to have a loud holosystolic murmur on auscultation heard over the entire precordium loudest in the apical area on day two. As TTE showed severe MR with mildly dilated left atrium and ventricle, he was characterized as acute MR given no changes in chamber sizes. However, the patient remained hemodynamically stable, with no signs of heart failure or cardiogenic shock. This asymptomatic clinical presentation of acute severe MR is rare. The cause was unidentified despite investigations such as cardiac catheterization and TEE showing normal results, with no history of rheumatic fever or evidence of amyloidosis. Consequently, it was thought to be due to degenerative mitral valve disease with mild myxomatous mitral valve resulting in sudden rupture of the posterior leaflet of chordae tendineae [11]. Hence, we referred it to as no snap as no mitral valve snap, no crackles due to the absence of crackles on lung examination, and pop due to sudden rupture of chordae tendineae.

## Conclusions

Overall, acute MR due to chordae rupture is a medical emergency that needs to be recognized and treated urgently to minimize significant morbidity and mortality. Optimizing patient outcomes requires early diagnosis and suitable treatment plans. We suggest that early recognition of acute MR in asymptomatic patients is associated with low intraoperative mortality and successful surgical intervention of 80-90%. Serial physical examinations are crucial and can aid in early diagnosis.
